# The Role of MDM2 in Promoting Genome Stability versus Instability

**DOI:** 10.3390/ijms18102216

**Published:** 2017-10-23

**Authors:** M. Reza Saadatzadeh, Adily N. Elmi, Pankita H. Pandya, Khadijeh Bijangi-Vishehsaraei, Jixin Ding, Christopher W. Stamatkin, Aaron A. Cohen-Gadol, Karen E. Pollok

**Affiliations:** 1 Department of Pediatrics (Division of Hematology/Oncology), Indianapolis, IN 46202, USA; msaadatz@iu.edu (M.R.S.); phpandya@iu.edu (P.H.P.); khbijang@iupui.edu (K.B.-V.); jixiding@iu.edu (J.D.); 2 Herman B. Wells Center for Pediatric Research, Indiana University School of Medicine, Indianapolis, IN 46202, USA; aelmi@indiana.edu (A.N.E.); cstamatk@iu.edu (C.W.S.); 3 Goodman Campbell Brain and Spine, Indianapolis, IN 46032, USA; acohenmd@gmail.com; 4 Herman B. Wells Center for Pediatric Research, Indiana University Simon Cancer Center, 1044 West Walnut Street R4 302, Indianapolis, IN 46202-5525, USA

**Keywords:** MDM2, p53, DNA damage, genome instability

## Abstract

In cancer, the mouse double minute 2 (MDM2) is an oncoprotein that contributes to the promotion of cell growth, survival, invasion, and therapeutic resistance. The impact of MDM2 on cell survival versus cell death is complex and dependent on levels of MDM2 isoforms, p53 status, and cellular context. Extensive investigations have demonstrated that MDM2 protein–protein interactions with p53 and other p53 family members (p63 and p73) block their ability to function as transcription factors that regulate cell growth and survival. Upon genotoxic insults, a dynamic and intricately regulated DNA damage response circuitry is activated leading to release of p53 from MDM2 and activation of cell cycle arrest. What ensues following DNA damage, depends on the extent of DNA damage and if the cell has sufficient DNA repair capacity. The well-known auto-regulatory loop between p53-MDM2 provides an additional layer of control as the cell either repairs DNA damage and survives (i.e., MDM2 re-engages with p53), or undergoes cell death (i.e., MDM2 does not re-engage p53). Furthermore, the decision to live or die is also influenced by chromatin-localized MDM2 which directly interacts with the Mre11-Rad50-Nbs1 complex and inhibits DNA damage-sensing giving rise to the potential for increased genome instability and cellular transformation.

## 1. Introduction

A number of mouse double minute 2 (MDM2) protein–protein interactions contribute to how eukaryotic cells sense, manage, and resolve DNA damaging events. MDM2 is a multi-functional protein, and the most studied function is its role as an E3 ubiquitin ligase. Indeed, the critical role of the well-documented MDM2-p53 connection in gauging genome integrity and promoting cell survival or death is underscored by the conservation of both proteins throughout the phylogenetic tree. By utilizing modeling approaches that focused on the homology of MDM2 and *Trichoplax* p53 domains, Srivasta et al. predicted that MDM2-p53 interactions existed even in simple organisms such as placozoans over a billion years ago [1]. Furthermore, the p53-independent role of MDM2 in promoting genome stability versus instability and the emergence of transformed cells due to MDM2 dysregulation are still in the early phases of investigation and will be discussed in this review. An interplay of MDM2 isoforms, MDM2 expression levels, and cellular context dictate whether genome stability or instability is the outcome of DNA damage.

## 2. Mouse Double Minute 2 (MDM2) Isoforms

MDM2 is one of the most highly regulated proteins due to its significant role in modulating p53-dependent and -independent functions in various cancers [2]. In humans, the *MDM2* gene was first isolated in 3T3DM cells from double minutes which are extrachromosomal amplified DNAs. In 3T3DM cells, the *MDM2* gene was amplified more than 50 times, and gene expression studies in NIH3T3 and Rat2 cells indicated that overexpression of MDM2 induced tumorigenesis in vivo [3]. Human MDM2, also referred to as human double minute 2 (HDM2), is located on chromosome 12q14.3–q15 [3,4] and overexpression and/or amplification of MDM2 has been observed in various human cancers and can contribute to genomic instability, thus, further promoting tumorigenesis [5,6]. The incidence of MDM2 gene amplification in human cancers is ~7% with soft tissue tumors, esophageal carcinomas and osteosarcoma typically having an increased frequency of MDM2 gene amplification [7,8]. 

MDM2 expression is regulated at the transcriptional level by two upstream open reading frames (uORFs) in the MDM2 gene which can mediate a switch in promoter selection [2,9]. Selection of the first promoter (P1) mediates transcription of the MDM2 gene where exon 2 is spliced out but the two uORFs remain. This generates the long MDM2 transcript (l-MDM2) [10] and translation of l-MDM2 produces a 90 kDa protein which is referred to as the MDM2-full length (MDM2-FL) protein. Selection of the second promoter (P2) results in the removal of the two uORFs but exon 2 remains resulting in the shortened MDM2 (S-MDM2) transcript and just like the l-MDM2 transcript, produces the 90 kDa MDM2-FL protein upon translation [2]. It is important to note that out of the 12 exons present in the *MDM2* gene, the first two exons (exon 1 and exon 2) do not encode for protein [11]. Rather, P1 is upstream of exon 1 and P2 is upstream of exon 2 [11]. Therefore, both l-MDM2 and S-MDM2 transcripts result in translation of the FL-MDM2 protein since the start codon is present around exon 3 [11,12]. Notably, the majority of the MDM2 protein is derived from the S-MDM2 transcript. In contrast to the l-MDM2 transcript, translation of the S-MDM2 transcript is 6-8 fold more efficient [10,12]. Furthermore, the S-MDM2 transcript has a low expression in normal cells but is highly inducible and overexpressed in a number of tumors [2], whereas, the l-MDM2 transcript is constitutively expressed in both normal and tumor tissues [2]. While factors that mediate transcriptional regulation at the P1 promoter on MDM2 still remain underexplored, it has been reported that oncoproteins such as the *N*-myc proto-oncogene (*MYCN*) can regulate MDM2 transcription through the P2 promoter leading to the overexpression of MDM2 in cancers such as neuroblastoma [10,13,14]. In addition, it is well documented that wildtype p53-mediated MDM2 transcription driven from the P2 promoter initiates a critical regulatory mechanism during DNA damage and repair. A major reason for decreased transcription driven from the P1 promoter is that wildtype p53 has only limited binding to P1 promoter of MDM2 compared to the P2 promoter [10,11,15,16]. As will be discussed in this review, the p53-mediated transcription and subsequent translation of MDM2-FL result in a feed-forward loop to regulate p53 levels after p53 has fulfilled its role in mediating cellular growth arrest, repairing DNA damage, or activating apoptosis. Thus, the outcome of the feed-forward loop is cytoprotective because it prevents accumulation of p53 when it is not required. MDM2-FL inhibits and promotes degradation of wildtype p53 resulting in normalization of p53 basal levels and cell survival [15].

Another factor that contributes to the observed MDM2 overexpression is a single nucleotide polymorphism (SNP) in the MDM2 gene at position 309 where the thymine is replaced by guanine (*MDM2* SNP309) [17]. Additionally, MDM2 transcripts and protein expression are elevated in cells with homozygous G/G MDM2 SNP309 [17]. Along with overexpression of MDM2, several MDM2 splice variants or isoforms can be overexpressed in various human cancers and normal tissues [17]. While the primary function of MDM2 as an E3 ubiquitin ligase refers to its targeting and inhibition of wild-type p53 via proteasomal degradation, this canonical MDM2 function may be modified or altered depending on the presence of MDM2 isoforms [5,18]. MDM2 isoforms can result from alternative splicing, aberrant splicing (splicing within the exons or introns), and through the initiation of transcription from two different promoter sites as described above [2,19]. The MDM2 isoforms could be expressed in normal tissues, as well as, in human cancers such as pediatric rhabdomyosarcoma, breast cancer, glioblastoma, and liposarcoma [5,19,20]. Notably, Chandler and colleagues reported that MDM2 isoforms could be specifically induced by genotoxic stressors such as UV irradiation or chemotherapy. Increased MDM2 isoforms have been observed in cisplatin-treated pediatric rhabdomyosarcoma [20,21]. Regardless of the factors that produce these variants of MDM2 in the context of cancers, overexpression of MDM2 isoforms in high-grade tumors have been correlated with poor prognosis [18]. Therefore, it is imperative to understand and further investigate these various MDM2 isoforms and the role they play in cancer.

Although recent scientific advancements have led to the identification of 72 diverse MDM2 isoforms in various human cancers and normal tissues, the functional roles for many of these spliced variants remain underexplored, and some isoforms lack the potential to be translated into proteins [5,22]. In contrast, the MDM2 isoforms MDM2-A (ALT2), MDM2-B (ALT1), and MDM2-C (ALT3) can be translated into proteins and have frequently been detected in various cancers [5,22]. 

**Figure 1:**
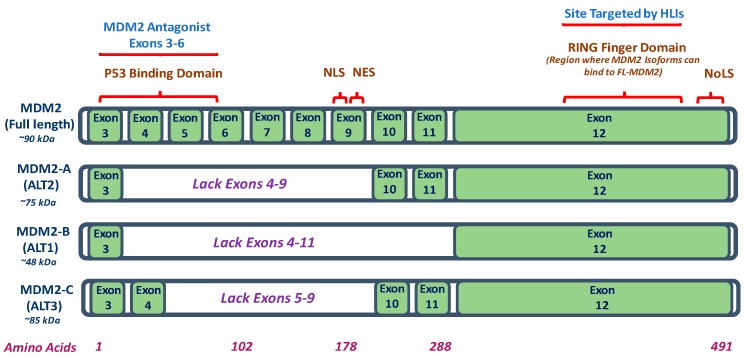
Illustrative Representation of MDM2-FL and MDM2 Isoforms (MDM2-A, MDM2-B, and MDM2-C). Adapted and modified from Huun et al. [5]. NES = nuclear export signal, NoLS = nucleolar localization signal, and NLS = nuclear localization signal.

The MDM2-FL contains several signaling and regulatory domains necessary for its function as depicted in Figure 1. To mediate localization into and out of the nucleus and cytoplasm, the MDM2 contains a nuclear export signal (NES), nucleolar localization signal (NoLS), and nuclear localization signal (NLS) (Figure 1) [5]. Additionally, MDM2-FL also contains a p53-binding domain and an E3 ubiquitin-ligase-mediating RING-finger domain that can bind to other MDM2-FL proteins at the N-terminus and C-terminus, respectively (Figure 1) [5]. However, MDM2 isoforms (MDM2-A, B, and C) differ from MDM2-FL in that they have several exons spliced out within their p53 binding domain located at the N-terminus and lack the amino acid domains for NLS and NES (Figure 1) [5,21]. On the contrary, the C-terminus RING-finger domain of these MDM2 isoforms is still present; therefore, these isoforms still preserve their ability to bind MDM2-FL but they lack the full capacity to target the wild-type p53 for proteasomal degradation (Figure 1) [5,21].

Interestingly, two MDM2 isoforms (MDM2-A and MDM2-B) have been reported to have both tumor-promoting and anti-tumor properties. MDM2-A (75 kDa), which lacks exons 4–9, activates p53 in vitro and decreases tumorigenesis (Figure 1) [5]. However, in vivo, it promotes tumor progression by increasing the expression of Cyclin D1 and Cyclin E which aid in cell cycle progression [5,23]. Similarly, MDM2-B (48 kDa), which is the most common MDM2 isoform observed in cancers, has exons 4–11 spliced out but is reported to promote tumor progression by inducing Cyclin D1 and Cyclin E in vivo [5]. The anti-tumor properties of MDM2-B are observed when it binds to MDM2-FL inhibiting the MDM2-FL-p53 interaction resulting in p53 stabilization/activation and cellular growth arrest [5].

In contrast, the function of MDM2-C (85 kDa), which lacks exon 5–9, remains fairly elusive even though it is frequently expressed in human cancers (Figure 1) [5]. Studies by Okoro and colleagues have indicated a p53-independent role for MDM2-C mediating cellular transformation in soft agar assays [17]. In MDM2 SNP309 estrogen receptor positive (ER^+^) breast cancer cell lines treated with estrogen, there was an increase in MDM2-C expression in the nucleus and cytoplasm, but this did not lead to degradation or decreased p53 levels [17]. Since MDM2-C lacks the NLS, it is presumably imported into the nucleus along with MDM2-FL which contains a NLS (Figure 1). Along with the detection of MDM2-C in ER^+^ breast cancer cell lines, Okoro et al. found increased MDM2-C expression in Burkitt Lymphoma cells (MANCA) and an osteosarcoma cell line (SJSA-1) [17]. Additional studies designed to elucidate the functional properties and biological effects of MDM2-C in other cancers as well as relationships between other isoforms need to be conducted in the future. 

Along with the molecular and structural differences observed between MDM2-FL and its isoforms, they also differ in their subcellular localization. Studies by Huun and colleagues on MDM2 isoforms in breast cancer cells reported that approximately 85% of MDM2-FL were located exclusively in the nucleus, whereas, MDM2-A, -B, and -C were located in both the cytoplasm and nucleus [5]. Since MDM2-A, -B, -C do not have a NLS (all lack exon 9), they presumably are shuttled into the nucleus via MDM2-FL. The location of these MDM2 isoforms depends on the cell type and its environment as seen in the case of MDM2-B which can be found exclusively in the cytoplasm of breast cancer cells and lung carcinomas, but is located in the nucleus of mouse embryonic fibroblasts [5]. All MDM2 isoforms studied to date can co-localize with MDM2-FL and prevent MDM2-FL from inhibiting wild-type p53, thereby, increasing wild-type p53 function [19]. However, despite the ability of MDM2 isoforms described above to promote wild-type p53 function in some instances, the presence of MDM2 isoforms can also promote tumorigenic properties [19]. Promotion of tumorigenesis can be attributed to the fact that MDM2 and its isoforms also play a role in noncanonical p53-independent mechanisms that regulate the cell cycle, DNA repair, and cell differentiation [24,25]. One such role of MDM2 is that it interacts with the polycomb repressive complex 2 (PRC2) via direct interaction with EZH2. EZH2 is the active component of the PRC2 complex and represses target gene expression through trimethylation of histone 3 at lysine 27 (H3K27me3) [26]. MDM2 enhances the histone trimethylation activity of the PRC2 complex. The extent to which various MDM2 isoforms may interact with and regulate the PRC2 complex is not known [26]. Furthermore, expression of MDM2 isoforms can regulate mutant p53 levels in cancer cells (Table 1).

Zheng et al. demonstrated that increased levels of MDM2 isoforms correlated with increased mutant p53 in tumors. The MDM2 isoform MDM2-B promoted mutant p53 accumulation in cancer cells thereby increasing tumorigenesis [18]. Moreover, MDM2-B could interact with MDM2-FL which inhibits the ability of MDM2-FL to target and degrade mutant p53 in various cancer cell lines. Additionally, while MDM2-FL could target both wild-type and mutant p53 for degradation, MDM2-FL-mediated degradation of wild-type p53 meant that wild-type p53 was no longer available to induce expression of MDM2-FL. Thus, mutant p53 accumulated [18].

One of the most studied functions of MDM2 in regards to p53 regulation is its RING finger-dependent E3 ubiquitin ligase activity [27,28,29]. MDM2 directly interacts with p53, facilitates the transport of p53 from the nucleus to cytoplasm, and monoubiquitinates, or in some instances, multi-monoubiquitinates p53 [30]. The subsequent poly-ubiquitination of p53 required for degradation by the 26S proteasome is provided by other E3 or E4 ligases such as UBE4B [31]. The functional outcome of increased p53 degradation is to promote cell survival and p53-independent roles of MDM2 in promoting survival are discussed below. Also, following DNA damage, MDM2 is phosphorylated on Ser395 and Ser407 which increases the ability of MDM2 to ubiquitinate itself [28,32] which promotes MDM2 degradation [33]. MDM2 also binds to and inhibits the transcriptional activity of p53 family member p63 and p73. For detailed information on the interactions of MDM2 and other E3 ligases with the p53 family members, see the reviews [27,28,29,34,35,36,37,38,39].

During evolution, it is believed that a duplication event at the MDM locus occurred with MDM2 and MDMX (also referred to as MDMX4) emerging as the central negative regulators of p53. It has been proposed that around the time of the duplication event, MDM2 obtained its E3 ligase activity while MDMX was preserved to regulate MDM2 ligase stability via heterodimerization with MDM2 [40]. MDM2 and MDMX share 31% homology at the amino acid level with both proteins containing a domain that interacts with p53 as well as comparable acidic, zinc finger, and RING domains. MDMX and MDM2 interact via their RING domains resulting in a MDMX-dependent increase in MDM2-mediated mono-ubiquitination of p53 [41]. Most notably, amplification of the *MDMX* gene is found in 10–25% of breast cancers, soft tissue tumors, and in tumors of the central nervous system [42,43]. In cancers where gene amplification is associated with increased MDMX protein expression, this would presumably increase the MDM2 E3 ligase activity leading to downregulation of p53 protein and increased survival of cancer cells [44]. 

## 3. Role of DNA Damage in Stability and Modification of MDM2

**Figure 2:**
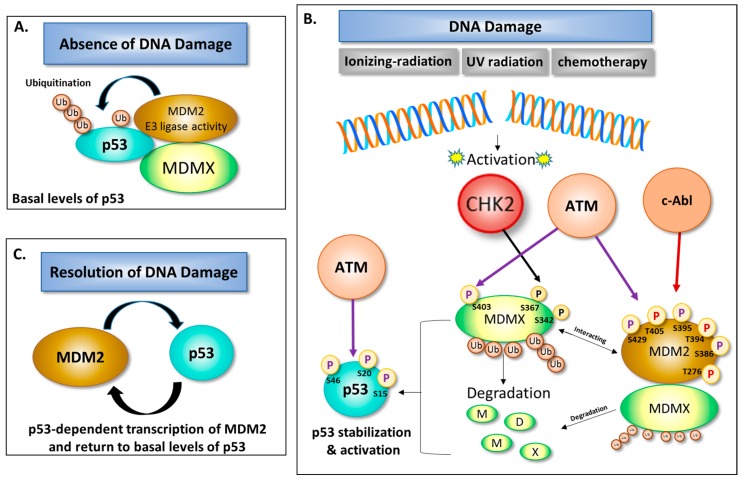
Regulation of p53 by MDM2 and MDMX following DNA damage. (**A**) In the absence of DNA damage, p53 levels and activation are tightly controlled by the MDM2-MDMX complex, and (**B**) upstream kinases act as sensors of DNA damage to regulate MDM2, MDMX, and p53; (**C**) Upon resolution of DNA damage, the p53/MDM2 feedback loop controls p53 levels. Ub = ubiquitin, P = phosphorylation site (purple arrow = phosphorylation by ATM; red arrow = phosphorylation by c-Abl; black = phosphorylation by CHK2); S = serine, and T = tyrosine.

The universal response of wildtype p53 cells to DNA damage induced by ionizing radiation (IR), ultraviolet irradiation, and cancer therapeutic agents such as platinum, intercalating, and DNA alkylating/methylating agents [45] is to activate the tumor suppressor p53 protein (Figure 2). The cellular response to DNA damage is primarily governed by the PI3K-related serine/threonine kinases (PIKKs). PIKKs are an important component of the DNA damage response which upon activation can phosphorylate both p53 and MDMX as well as a variety of proteins involved in DNA repair mechanisms, tolerance to DNA damage, cell cycle checkpoint activation, as well as induction of apoptosis, or senescence [46]. In response to DNA damage, the PIKK CHK2 can directly phosphorylate p53 (residues T18 and S20) and MDMX (residues S367 and S342) leading to p53 accumulation and apoptosis [47]. To regain genomic stability and prevent transformation following DNA damage, p53 is activated. A number of post-translational modifications of key regulatory proteins are involved in resolving DNA damage or promoting cell death. Following DNA damage, MDMX is phosphorylated on S403 by Ataxia Telangiectasia Mutated (ATM) and on residues S342 and S367 by Chk2 (cell cycle checkpoint regulator and putative tumor suppressor); phosphorylated MDMX is then degraded by MDM2 preceding activation and accumulation of p53 [48,49]. Pereg et al. also showed that decreasing levels of MDMX by RNAi increased p53 response to DNA damage as well as the loss of ATM inhibited both MDMX degradation and p53 stabilization. Mutation of MDMX on these specific residues (S403, S342 and S367) conferred resistance to subsequent MDM2-mediated ubiquitination and degradation of MDMX. Chen et al. also showed that phosphorylation of MDMX at S342 and S367 require Chk2 kinase activity and Chk2 stimulates MDM2-dependent ubiquitination and degradation of MDMX. Therefore, after DNA damage, the E3 ligase activity of MDM2 is redirected to some extent from p53 to MDMX which contributes to p53 activation [49]. The c-Abl tyrosine kinase is also activated by a variety of DNA damaging agents [50], and is another key regulator of MDM2. In vitro studies have shown that c-Abl phosphorylates human Mdm2 on Tyr276 as well as Tyr394 and Tyr405. The outcome of c-Abl-mediated phosphorylation of Mdm2 at Tyr394 impairs the ability of Mdm2 to inhibit p53 stabilization, activation, and p53-mediated apoptosis [51,52]. Furthermore, it has been proposed that c-Abl phosphorylation of Mdm2 increases Mdm2-MdmX binding thereby increasing Mdm2-directed MdmX ubiquitination. As a result, an increase in MdmX ubiquitination ultimately destabilizes the Mdm2-MdmX complex, promoting p53 stabilization [53] (Figure 2). Pan et al. demonstrated that promotion of MDMX ubiquitination and degradation by MDM2 was ARF protein dependent (ARF tumor suppressor, p14^ARF^ in human or p19^ARF^ in mouse) and required the N-terminal domain of ARF protein. It is important to emphasize that the N-terminal domain of ARF can also inhibit MDM2 ubiquitination of p53 [54]. 

MDM2 is overexpressed in a variety of different types of cancers which is due to its gene amplification or regulation at the protein level [55]. While the role of MDM2 as a negative regulator of p53 has been widely studied, more recent studies are emphasizing the p53-independent role of MDM2 in cell cycle progression, DNA damage response and apoptosis [56,57,58]. Senturk et al. previously demonstrated that osteosarcoma cell lines exhibited resistance to doxorubicin and etoposide due to overexpression of MDM2 (~20–50 fold amplification of the *MDM2* gene). The cells were wildtype p53, and the function of overexpressed MDM2 in their study was shown to be p53 independent. These observations could have important implications for the choice of chemotherapeutic agents in the treatment of Mdm2-overexpressing tumors [59]. It has also been reported that in some estrogen-receptor positive (ER+) breast cancer cells, that the signaling network of ER-MDM2-RB-E2F1 is an essential pathway for estrogen-mediated p53-independent signal transduction. Furthermore, ER-mediated overexpression of MDM2 due to the activity of estrogen in both wild type (MCF7) and mutant (T47D) p53 cells was p53-independent [60]. Kim et al. suggested that Mdm2 plays a role in enhancing ERα-mediating gene expression and estrogen-responsiveness through interactions with ERα in breast cancer cells [61].

## 4. The Balancing Act of MDM2 and p53 Expression—Implications for Cell Survival

**Figure 3:**
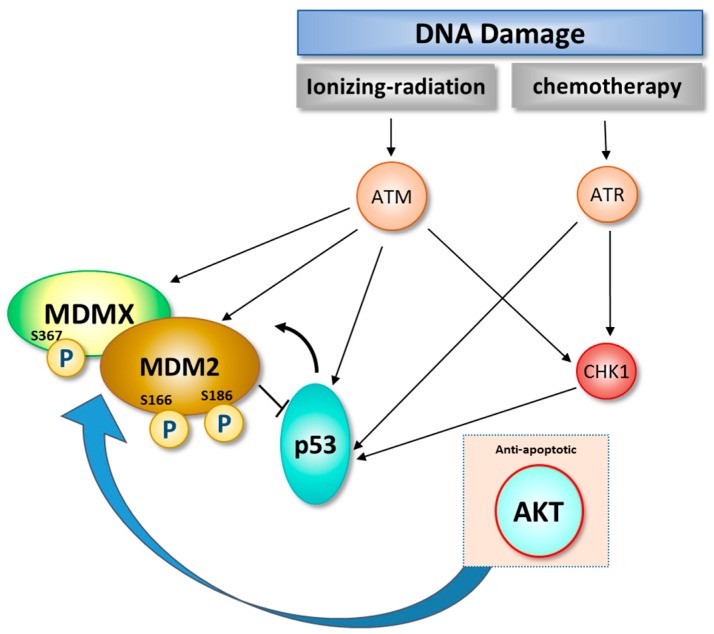
Regulation of the MDM2-signaling network by the pro-survival kinase AKT. AKT-mediated phosphorylation of Mdm2 promotes Mdm2 entry into the nucleus and enhances its ubiquitination-promoting function which leads to p53 inactivation, inhibition of apoptosis, and increased survival. Phosphorylation of MDMX by AKT stabilizes MDM2 E3-ligase activity.

The relationship between p53 and MDM2 impacts every cellular biological system associated with cell development, growth control, and apoptosis [62,63]. Experiments utilizing knock-out mice have revealed that deletion of the MDM2 gene results in embryonic lethality since p53 levels can no longer be regulated and therefore increases. However, lethality in MDM2 gene knock out mice can be reversed by co-deletion of the *p53* gene, further emphasizing how an imbalance of MDM2 and p53 can dramatically affect survival at the cellular and organism levels [64,65]. It has been advantageous to the survival of multicellular organisms to select for multiple mechanisms that control the balance between MDM2 and p53 [33]. Following DNA damage from endogenous insults or the environment, p53 is phosphorylated on a number of amino acid residues, such as Ser15, 20, 33 and 37, and phosphorylation on N-terminal Ser15 and -37. Once p53 is phosphorylated at these residues, Mdm2 can no longer interact with p53 [66,67]. MDM2 function and subcellular localization can be controlled to some degree by the pro-survival kinase, AKT (Figure 3). Activated AKT phosphorylates MDM2 on Ser186 and Ser166 which in turn enhances the ubiquitination function of Mdm2 and subsequently causes reduction of p53 protein levels. Furthermore, AKT-mediated phosphorylation of MDMX at Ser367 also stabilizes MDM2 E3-ligase activity [68]. AKT-mediated phosphorylation of MDM2 promotes the subcellular localization of Mdm2 from the cytoplasm to the nucleus [68,69,70,71].

As mentioned above, the MDM2-p53 connection is tightly regulated via a feedback loop between the proteins. As DNA damage increases, p53 is stabilized, and p53- mediated transcriptional activation of downstream target genes including the MDM2 and pro-apoptotic genes ensues [72]. Also, the p53–MDM2 relationship provides critical oversight of complex signaling networks involving Wnt, Ras, Retinoblastoma protein (Rb) and Myc [33]. Moreover, the intricate regulation of MDM2 at the transcriptional, post-transcriptional, and post-translational levels all contribute to the levels of MDM2 expression and activity; the well-studied MDM2 regulators are summarized in Table 2. The vast majority of MDM2 targets are ubiquitinated by Mdm2 and targeted for proteasomal degradation (Table 3). The consequence of MDM2 binding to critical signal transduction molecules is summarized in Table 3. These tables were adapted and modified from the review by Riley and Lozano [14].

## 5. p53-Independent Role of MDM2 in Genome Instability and Survival

The characterization of MDM2 as a p53 inhibitor is widely accepted in the scientific realm, however current studies suggest that MDM2 possesses functions independent of p53, which also appear to influence tumorigenesis [82]. MDM2 overexpression has been detected in numerous human cancers that also bear mutant p53 or lack p53 [83,84]. For instance, human sarcomas and bladder cancers showed an overexpression of MDM2 in addition to mutated p53. Patients with the cancers above and whose tumors possessed both abnormalities had decreased survival compared to patients with either abnormality alone [83,85]. Mouse studies also support the p53-independent role for MDM2 in tumorigenesis. In Eµ-myc transgenic mice models, approximately 30% of the lymphomas with deleted or mutated p53 expressed MDM2. Studies in mice that lacked p53 further elucidated the independent role of MDM2 in tumorigenesis. In contrast to mice with p53 deletion, tumors found in p53 null mice that were heterozygous for MDM2 or overexpressed MDM2 had a different composition of tumor types [86,87]. The MDM2 transgenic mice further exhibited ploidy, which is a marker for genomic instability regardless of p53 expression. These studies and many others corroborate the hypothesis that MDM2 has p53 independent oncogenic functions and elucidation of these features will help explain how MDM2 contributes to genome instability [86,87].

**Figure 4:**
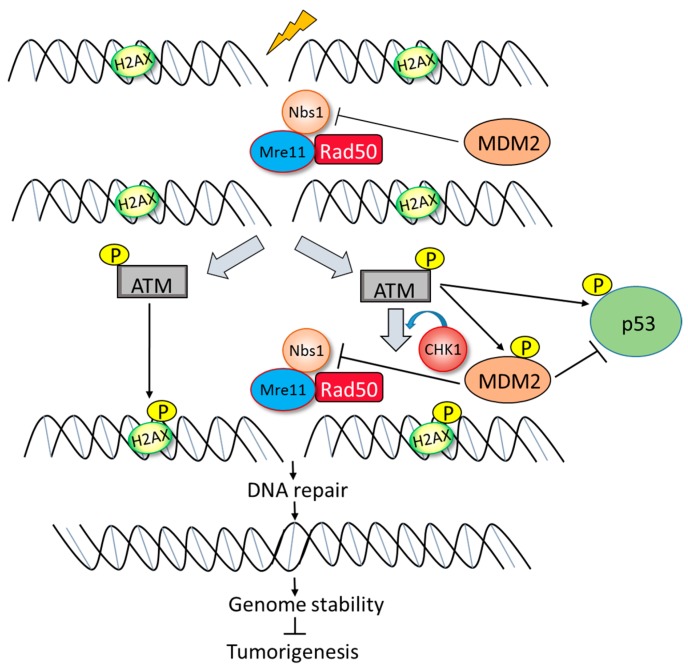
Model of inhibition of DNA break repair by Mdm2. Double-strand break induced by irradiation is detected by the M/R/N complex. ATM is recruited to the DNA-strand break and activated by auto-phosphorylation. In turn, activated ATM phosphorylates Nbs1, histone H2AX, p53, and Mdm2. MDM2 delays the early phosphorylation process, which is necessary for DNA double-strand break repair, causing inefficient DNA break repair and if not repaired could lead to genomic instability and tumorigenesis (Modified from Bouska and Eischen [56,84,93]). Inhibition = 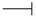 Activation = 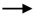.

The underlying mechanisms on how MDM2 promotes genome instability are starting to be uncovered (Figure 4). A direct physical interaction between MDM2 and Nbs1, a component of the Mre11-Rad50-Nbs1 (M-R-N) complex was identified [56,73,84]. As background, the M-R-N complex is essential for maintaining DNA integrity during double-strand break repair, meiotic recombination and telomere maintenance [73]. Nbs1 is believed to promote the localization of the M-R-N complex to DNA damage sites, while Rad50 is a “structural-of-chromosome” family member with ATP motifs that provide energy to the M-R-N complex. Rad50 also tethers DNA ends together, while Mre11 processes the DNA breaks with exonuclease and endonuclease activity [74]. These proteins are essential for survival, as deletion of any of the three proteins in mice is embryonic lethal [75]. In humans, a mutation in Nbs1 leads to the development of Nijmegen breakage syndrome and ataxia-telangiectasia-like disorder (ATLD). Patients with ATLD display genomic instability, while those with Nijmegen breakage syndrome have a very high incidence of cancer [73,88]. Following DNA damage, the M-R-N complex promotes the phosphorylation of ataxia-telangiectasia mutated (ATM), a kinase responsible for signaling DNA damage [89,90].

Following DNA damage, ATM phosphorylates histone H2AX, which is essential for the long-term retention of repair factors at sites of DNA breaks [91] (Figure 4). Once this response occurs, proteins such as ATM and the M-R-N complex re-localize to damaged sites and form nuclear foci. Once the DNA damage is repaired, the nuclear foci resolve. While the exact mechanisms are not understood, MDM2 appears to regulate the ability of the M-R-N complex to signal that DNA damage has occurred. Bouska et al. postulated that MDM2 interferes with the function of Nbs1 during early signaling events following DNA damage. Their study indicated that high levels of MDM2 delayed the phosphorylation of H2AX, and thus ATM signaling which results in a delay in DNA repair. This delay in foci formation was due to the high levels of MDM2 interacting with Nbs1 and ultimately delaying the repair of DNA breaks [56]. These studies are consistent with the concept that MDM2 contributes to genome instability and tumorigenesis by the delay of DNA breakage repair in a p53-independent manner. While the role of MDM2 isoforms in DNA damage/repair mechanisms still requires further investigation, it can be speculated that the three MDM2 isoforms previously mentioned (MDM2-A, MDM2-B, and MDM2-C) may not be responsible for the delay in DNA repair by the same mechanisms as observed through the interaction of FL-MDM2 with the M-R-N complex (specifically Nbs1). This speculation can be attributed to the fact that interaction between FL-MDM2 and Nbs1 requires the presence of amino acids 198–314 which spans exon 9 through exon 12 on FL-MDM2 (Figure 1) [5,73]. Notably, MDM2 isoforms (MDM2-A, B, C) all lack exon 9. Therefore, MDM2 isoforms are unable to properly bind Nbs1 for delaying DNA repair via M-R-N complex. Though, due to various other factors that play a role in DNA damage/repair pathways, it is quite possible that future studies will elucidate novel mechanisms by which MDM2 isoforms regulate DNA damage/repair that may or may not be dependent on p53 activation. Persistence of chemotherapy-induced DNA damage has been observed in wild-type p53 glioblastoma cells [92] as well as p53 deleted or mutant p53 ovarian cells [6] treated with the first-generation MDM2 antagonist Nutlin3a and this correlated with increases in MDM2 expression and cell death. Also, while genetic analyses have demonstrated that the main target for both MDM2 and MDMX is p53, MDMX also possesses p53-independent roles similar to MDM2 [44,58,63]. When MDMX is overexpressed in tumor cells, it can influence genome instability through the regulation of p53 [6] as well as inhibit the DNA damage response which would increase genome instability and transformation independent of p53 and MDM2 [93].

Previous studies have suggested that normal cells lacking MDM2 can only survive if p53 is also absent. However, a study by Feeley et al. refutes this view and uses conditional MDM2 knock-out mouse modeling approaches to provide compelling evidence illuminating that MDM2 expression is required for growth and survival of p53 null lymphoma and sarcoma in vivo [82]. The results from the Feeley study elucidated that deletion of MDM2 in p53 null cells promotes events typically driven by p53-dependent cell cycle arrest and apoptosis. This elegant study further demonstrated that these events were dependent on the p53 family member, p73 [82]. This revelation was evident not only in cancer cells but also in normal fibroblasts. Furthermore, the study by Feeley and colleagues emphasized that the inhibition of MDM2 binding to p53 family members by MDM2 antagonists versus MDM2 deletion are both deleterious to the cells but arrive at death via different mechanisms [82].

## 6. Therapeutic Considerations and Conclusions

The integration of signals emanating from the multi-faceted MDM2 signaling network dictates to a large extent, whether cells commit to cell death or survival. Genome instability is a characteristic associated with almost all human cancers, but the p53-independent role of MDM2 in promoting mutational burden versus cell death requires further investigation. The Mdm2 protein is an emerging therapeutic target for treatment of cancers [55]. Importantly, in anti-cancer therapies that will modulate MDM2 function, it will be critical to ensure that chromatin-bound MDM2 prevents repair of therapy-induced DNA damage that results in cell death and not the emergence of resistant tumors with an increased mutational burden. As mentioned previously in this review, Mdm2 can be overexpressed due to gene amplification or polymorphisms that increase promoter activation in many cancers activation [55]. Analysis of patient specimens from multiple solid tumors indicates that MDM2 protein is elevated and correlates with poor prognosis [8,55]. Furthermore, the induction of the MDM2-p53 autoregulatory loop by frontline cytotoxic agents provides fertile ground to develop novel anti-cancer targeted therapies that sustain p53 activation and increase cancer-cell sensitivity to lower doses of cytotoxic therapy. Over the past decade, an entirely new field of MDM2 protein–protein interaction inhibitors (PPIs) has emerged [94]. The sustained activation of p53 by use of MDM2 PPIs in the context of cytotoxic chemotherapy remains an active area of study and clinical trial development (Table 4) [95]. The PPIs target the hydrophobic core of MDM2/MDMX and restore the normal active conformation of p53 (Table 4). For detailed information regarding clinical trials of MDM2/MDMX inhibitors, refer to the reviews by [95,96]. Initial clinical trials that utilize MDM2 PPIs have reported evidence of therapeutic responses in relapsed leukemia [97] and liposarcoma [98]. 

There are a number of pre-clinical studies demonstrating the promise of targeting MDM2 via PPIs in cancers with either high endogenous MDM2 expression or targeting MDM2 following induction of MDM2 expression via DNA damaging agents [92,107,108,109,110,111,112]. MDM2 antagonists bind to the N-terminal hydrophobic pocket in Mdm2 located in exons 3–6 (Figure 1), and depending on the MDM2 antagonist utilized can block interactions of Mdm2 with p53 but also other critical signaling proteins such as p73α, E2F1, and HIF1α [108,113,114,115,116,117,118]. Blocking these protein–protein interactions can lead to activation of p53/p73-mediated apoptosis and inhibition of HIF1α-mediated VEGF production, angiogenesis, invasion, and metastasis [113,114,115,116,117]. Importantly, since MDM2 antagonists bind to the N-terminal pocket (exons 3–6) of MDM2-FL to block p53 binding, these inhibitors will likely not directly affect other MDM2 isoforms such as MDM2-A (lacks exons 4–9), -B (lacks exons 4–11), and -C (lacks exons 5–9). In contrast, therapeutics such as the MDM2 ligase inhibitors (HLIs) that bind to the C-terminus of MDM2 are capable of not only affecting FL-MDM2 but also various MDM2 isoforms due to the presence of an intact C-terminus [119,120]. There are two subsets of the HLI family of inhibitors, the HLI98s and its homologue family known as the HLI373s. Both inhibitors act by inhibiting the E3 ubiquitin ligase activity mediated by the RING finger domain on the C-terminus of MDM2 [120,121]. However, they differ in that HLI98s are classified as 7-nitro-10-aryl-5-deazaflavins cell-permeable inhibitors, whereas, HLI373s are 5-dimethylaminopropylaminos but with a side chain that lacks the 10-aryl group [120,121]. Additionally, HLI373 is more potent compared to the HLI98s. Due to the blocking of the E3 ubiquitin ligase by these HLIs, the MDM2 proteins are not able to ubiquitinate wildtype p53 and target it for degradation [120]. Therefore, wildtype p53 protein accumulates, p53 transcriptional activity is induced, and it can mediate its tumor suppressive functions previously described in this review [120]. It has been reported by Kitagaki and colleagues that HLIs result in increased wildtype p53 levels in both transformed and non-transformed cells but promote apoptosis only in transformed cells expressing wildtype p53 [120]. Unlike the effect of HLIs on FL-MDM2, the impact of HLIs on MDM2 isoforms requires further exploration, but it is possible that these HLIs could still target the various MDM2 isoforms that have the full-length RING finger domain and E3 ubiquitin ligase activity. As suggested by Okoro and colleagues, whether these MDM2 isoforms retain the E3 ubiquitin ligase activity remains elusive and requires further study [17]. One can further speculate that since the RING finger domain in the C-terminus of MDM2 is the site for MDM2 isoforms to bind to FL-MDM2 as well as the site where these HLIs target (Figure 1), it is possible that treatment with these HLIs can actually impair the ability of MDM2 isoforms to bind to FL-MDM2. Thus, inhibiting their interaction with FL-MDM2 will result in free FL-MDM2 capable of inhibiting wildtype p53 through suppression of its transcriptional activity and by its ubiquitin-mediated proteasomal degradation. However, treatment with HLIs would also inhibit the E3 ubiquitin-ligase activity on the FL-MDM2 which will protect the wildtype p53 from degradation, result in its accumulation, and preserve its tumor suppressive functions.

Another strategy is to target MDM2 and its interaction with mRNA that encodes the X-linked inhibitor of apoptosis protein (XIAP) [122]. Binding of the MDM2 protein to the internal ribosome entry site (IRES) on the mRNA encoding the anti-apoptotic XIAP results in stabilization of MDM2 protein and increased translation of XIAP [123]. This regulatory mechanism is not dependent on p53 status, and the ability to modulate two independent pathways by disrupting a single interaction could be a benefit of this novel therapeutic approach [123]. Using a fluorescence polarization assay for high throughput screening of chemical libraries, Gu et al. identified a panel of inhibitors that block protein-RNA interaction (MDM2-XIAP) leading to MDM2 degradation [122]. In fact, in vitro cancer cell apoptosis and in vivo proliferation inhibition occur due to both inhibition of XIAP expression and also p53 activation following compound-induced downregulation of MDM2. Two of the MDM2-XIAP inhibitors are MX69 and MX3. MX69 binds to MDM2 and prevents the protein from interacting with the IRES of XIAP mRNA which results in decreased XIAP translation and MDM2 degradation. The authors also show that MX69 has no effect on normal human hematopoiesis cells in vitro and is well tolerated in animal models [122,123] while MX3 showed increased heart tissue damage, elevation of alanine aminotransferase (ALT), and decreased white blood cells in an animal model [122].

As described above, there are a number of therapeutic strategies that block the function of MDM2 and promote tumor cell death. Efficacy of the first-generation MDM2 antagonist Nutlin-3a in cancers such as liposarcomas that express MDM2-B has been investigated, however, responses were heterogeneous with no promising results of Nutlin3a treatment achieved in MDM2-B expressing liposarcomas [124]. For approaches employing MDM2 antagonists to be successful in the clinic, it will be important to understand how disruption of MDM2-FL-p53 interactions also impact MDM2 isoforms interactions with MDM2 as well as other MDM2 protein–protein interactions. Moreover, since MDM2-FL and other isoforms can modulate mutant p53 stability, it is possible that MDM2 antagonists could promote stabilization of mutant p53. In mutant p53 triple-negative breast cancer cells, however, we did not see increased stability of mutant p53 in cells treated with the MDM2 antagonist, Nutlin3a [108]. It is worth noting, however that a variety of cancer types that express MDM2-FL and different MDM2 isoforms still undergo cell death when exposed to MDM2 antagonists. Whether MDM2 isoforms attenuate this response will require further study.

There is evidence that MDM2 antagonists can preferentially increase p53-mediated apoptosis in cancer cells compared to normal cells. However, the overall impact of MDM2 antagonists on the ability of MDM2 to ubiquitinate other target proteins requires further investigation since this could affect tumor cell response to the MDM2 antagonists [125]. We and others have shown that in human xenograft models, exposure to MDM2 antagonists as single agents or in the context of standard-of-care therapy was well tolerated. For example, in an orthotopic model of recurrent GBM, we showed that MDM2 inhibition decreased the resistance to the standard-of-care agent temozolomide by sustaining p53 activation and blocking DNA repair [92]. Importantly, there was no decrease in complete bone marrow counts or bone marrow cellularity beyond the transient effects of temozolomide (TMZ) on the bone marrow, even when temozolomide was delivered at maximally tolerated doses [92]. Moreover, in a model of metastatic breast cancer, while MDM2 blockade potentiated the effects of carboplatin on inhibiting tumor growth, the therapy was well tolerated [92,101,108,118,119,126,127,128,129,130]. Complete blood counts, bone marrow cellularity, as well as the frequency of bone marrow-derived hematopoietic progenitor cells did not exacerbate carboplatin-mediated effects in vivo [131,132]. We and others have shown that toxicity and safety testing in mouse models may not always predict what is observed in humans so large animal studies will likely be more predictive. In a human liposarcoma study by Ray-Coquard et al., use of the MDM2 antagonist RG7112 led to thrombopoiesis in some of the patients [98]. In a follow-up study, Iancu-Rubin et al. demonstrated that exposure to RG7112 in rats and non-human primates led to decreased platelet counts but the effects of RG7112 exposure could be reversed once therapy stopped [131]. This data emphasizes that careful attention to scheduling of MDM2 antagonists will be critical in the clinical setting to avoid toxicity to sensitive cells such as those located in the bone marrow and the peripheral blood. Intermittent dosing regimens and modeling approaches for the use of MDM2 antagonists have been proposed by Higgins et al. [132]. 

Based on studies to date, MDM2 and its family member MDMX are powerful players that dictate how cells respond to DNA damage and stress. In this review, we have highlighted how MDM2 is regulated during DNA damage, and how chromatin-bound MDM2 or MDMX may contribute to cell death or survival. If a damaged cell can tolerate genome instability induced by MDM2 or MDMX, then there is the potential to promote cellular transformation. A concern with many cancer therapeutics is the adaptive tumor response to therapy, recurrent tumor growth, and the possibility of inducing secondary malignancies. The same concerns hold true for MDM2 antagonists since there is a window of opportunity to increase genome instability. However, once DNA damage surpasses a particular threshold, genome instability is too high and results in cell death. If MDM2 is to be successfully targeted in the clinic, it will be essential to learn how to best use MDM2 antagonists in combination with DNA-damaging agents and possibly other small molecule inhibitors that target intersecting pathways. Indeed, an interplay of MDM2 isoforms, MDM2 expression levels, and cellular context will dictate whether genome stability or instability is the outcome of anti-cancer therapies that target MDM2.

## Figures and Tables

**Table 1:** Presence of MDM2 isoforms and p53 status [5,10,17,23].

MDM2 Isoform	MDM2-A	MDM2-B	MDM2-C
**Model In Vitro and/or In Vivo**	MDM2-A transgenic mice; MDM2-A expressing transgenic mouse embryonic fibroblasts (MEFs); and, MDM2-A retrovirally transduced wildtype MEFs	Human lung cancer and colorectal cancer cell lines	Human breast cancer cell lines; human liposarcoma, breast carcinoma tissues, and osteosarcoma cells
**MDM2 Isoform Expression**	Increased expression of MDM2-A (75 kDa) in human cancer cells and/or tissues such as breast cancer and Hodgkin’s Lymphoma	Most common MDM2 isoform. Increased expression of MDM2-B (48 kDa) observed in in human cancers and/or tissues such as colorectal cancer, breast cancer, and Hodgkin’s Lymphoma	Increased expression of MDM2-C (85 kDa) in human cancer cells and/or tissues of breast cancer, osteosarcoma, and chronic myelogenous leukemia
**p53 Expression**	Accumulation of wildtype p53 activity	Accumulation of wildtype and mutant p53	p53 independent transformation function; does not function by inhibiting p53 transcriptional activity and does not show role in p53 degradation pathway
**Mechanism**	MDM2-A lacks wildtype p53 binding region but binds and sequesters FL-MDM2 to prevent FL-MDM2-dependent-degradation of wildtype p53	MDM2-B lacks the wildtype p53 binding domains but can interact with FL- MDM2 to prevent degradation of mutant p53	p53-independent function for cell proliferation; MDM2-C lacks p53 binding domain but exact mechanism requires further investigation

**Table 2:** MDM2 regulators and functional outcome [45,53,69,73,74,75,76,77,78,79,80].

Effectors of MDM2	Effect on MDM2 Function
PTEN	Transcriptional inhibition
NF-κB	Transcriptional activation
Raf	Transcriptional activation
Smad3/4	Transcriptional activation
E2F1	Transcriptional inhibition
ATM	MDM2 phosphorylation at S394 and/or S395 is required for p53 accumulation, stabilization and activation
c-AbI	Tyrosine phosphorylation of MDM2 facilitates MDM2-MDMX complex formation and regulates p53 stabilization
AKT	Phosphorylation of MDM2 at residues S166 and S188 inhibits its self-ubiquitination and at S186 Akt enhances the ubiquitination-promoting function of MDM2 which results in reduction of p53 protein
Daxx	Stabilizes; enhances interaction between Mdm2 & Hausp
Cyclin G	Dephosphorylation of Mdm2
MdmX	Inhibits auto-ubiquitination of MDM2 E3 ligase activity
Elf4/Mef	Transcriptional activation
p19ras	Blocks Mdm2-p73 interaction
Seladin-1	Blocks Mdm2-p53 interaction
RPS3/S7/S27	Blocks Mdm2-p53 interaction
L5/L11/L23/L26	Blocks Mdm2 ubiquitination of p53
p38	p300 binds to p53 and MDM2; there is evidence that p38 can phosphorylate p300 and increase capacity of MDM2 to promote p300 degradation.
Cyclin a-CDK complexes	phosphorylate MDM2 and affect interaction of MDM2 with proteins
p14^ARF^	E3 ligase inhibition in the context of MDM2-p53 interactionsE3 ligase activation in the context of MDM2-MDMX interactions
MTBP	Binds to MDM2 and Induces a G1 Arrest

PTEN = phosphatase and tensin homolog; NF-κB = nuclear factor κ-light-chain-enhancer of activated B cells; AKT = Protein kinase B is a serine/threonine-specific protein kinase; MTBP = MDM2-binding protein.

**Table 3:** Downstream targets of MDM2 [14,56,81].

Targets of MDM2	Result of Interaction with MDM2
p53	Decreases p53 activity
p73	Decreases p53 activity
p63	Decreases p53 activity
HDAC	Mdm2-HDAC interaction facilitates p53 acetylation
Nbs1	Inhibition of double strand break repair
β2 Androgen receptor	Ubiquitination and degradation via Akt/Mdm2
RB	Inhibits RB binding to E2F1
ATF3	Ubiquitination and degradation
E-cadherin	Ubiquitination and degradation
NF-κB/p65	MDM2 induces NF-κB/p65 expression transcriptionally through Sp1-binding sites
Chk2	Ubiquitination and degradation
NUMB	Alters subcellular localization; Ubiquitination and degradation

HDAC = Histone deacetylases; RB = retinoblastoma; E-Cadherin = A transmembrane protein that links plasma membranes of adjacent cells together in a Ca^2+^-dependent manner; aids in maintaining the rigidity of the cell layer; NF-κB/p65 = a subunit of NF-κB transcription complex; NUMB = Endocytic Adaptor Protein.

**Table 4:** List of MDM2-p53 inhibitors in previous or current clinical trials are listed MDM2 Inhibitors in Clinical Trials [99].

Compound Developer	Clinical Trial Phase and Status	References
RO5045337/RG7112	phase I	[100]
MDM2 antagonist	Completed	
(Roche)		
RO5503781/RG7388/Idasanutlin	phase I	[101]
MDM2 antagonist	Completed	
(Roche)		
AMG232	phase I	[102]
MDM2 antagonist	Completed	
(Amgen)		
CGM097	phase I	[103]
MDM2 antagonist	Ongoing but not recruiting	
(Novartis)		
DS-3032b/Benzodiazepinedione	phase I	[104]
MDM2 antagonist	Recruiting participants	
(Daiichi Sankyo)		
SAR405838	phase I	[105]
MDM2 antagonist	Completed	
(Sanofi S.A.)		
MK-8242/SCH 900242	phase I	[106]
MDM2 antagonist	Terminated	
(Merck)		
ALRN-6924	Phase I/2a	[94]
MDM2/MDMX dual antagonist	Ongoing recruiting	
(Aileron Therapeutics)		
